# The limits to equivalent living conditions: regional disparities in premature mortality in Germany

**DOI:** 10.1007/s10389-017-0865-5

**Published:** 2017-11-18

**Authors:** Thomas Plümper, Denise Laroze, Eric Neumayer

**Affiliations:** 1 Department of Socioeconomics, Vienna University of Economics and Business, Welthandelsplatz 1, 1020 Vienna, Austria; 2 Centre for Experimental Social Sciences, Universidad de Santiago de Chile, Concha y Toro 32C, 8340599 Santiago, Región Metropolitana Chile; 3 Department of Geography & Environment, London School of Economics and Political Science (LSE), Houghton Street, London, WC2A 2AE UK

**Keywords:** Premature mortality, Living standards, Health policy, Socioeconomic, Regional inequality

## Abstract

**Aim:**

Despite the country’s explicit political goal to establish equivalent living conditions across Germany, significant inequality continues to exist. We argue that premature mortality is an excellent proxy variable for testing the claim of equivalent living conditions since the root causes of premature death are socioeconomic.

**Subject and methods:**

We analyse variation in premature mortality across Germany’s 402 districts and cities in 2014.

**Results:**

Premature mortality spatially clusters among geographically contiguous and proximate districts/cities and is higher in more urban places as well as in districts/cities located further north and in former East Germany. We demonstrate that, first, socioeconomic factors account for 62% of the cross-sectional variation in years of potential life lost and 70% of the variation in the premature mortality rate. Second, we show that these socioeconomic factors either entirely or almost fully eliminate the systematic spatial patterns that exist in premature mortality.

**Conclusion:**

On its own, fiscal redistribution, the centrepiece of how Germany aspires to establish its political goal, cannot generate equivalent living conditions in the absence of a comprehensive set of economic and social policies at all levels of political administration, tackling the disparities in socioeconomic factors that collectively result in highly unequal living conditions.

## Introduction

Regional disparities in living conditions weaken the cement that holds nation states together. Welfare and transfer states such as Germany aspire to reduce such disparities. In fact, the German constitution, the *Grundgesetz*, espouses the explicit aim to achieve “equivalent living conditions” across German states (the *Länder*) and regions. Article 72 of the German basic law, which grants the federal government the right to interfere in the autonomy of the States “if and to the extent that the establishment of equivalent living conditions throughout the federal territory (…) renders federal regulation necessary in the national interest”, represents the culmination of a historical process toward greater fiscal redistribution, already built into the fiscal order of the Kaiserreich (1870–1918) and the Weimar Republic (1918–1933) as a principle.^1^ The Federal Republic of Germany relies on financial equalisation (*Finanzausgleich*), which redistributes tax revenue among Germany’s 16 states and provides poor states with additional financial contributions by the federal government.

This article demonstrates that fiscal redistribution and other transfer measures have not generated equivalent living conditions in Germany. Equivalence in living conditions cannot be directly measured. Instead, we use the propensity to die prematurely, defined as dying before the age of 75 years,^2^ as a powerful proxy for equivalence in living conditions, thus assuming that equivalent living conditions exist if premature mortality only varies stochastically. Systematic spatial patterns in premature deaths, in other words, indicate a diversion from the goal of equivalent living conditions. Accordingly, our findings of rather strong inequalities in premature mortality at the level of Germany’s districts (*Kreise*) and cities (*kreisfreie Städte*) suggest the persistence of unequal living conditions. Whilst on average approximately three out of four Germans reach their 75th birthday, the average citizen of Herne, a district in the old coal and steel region of North-Rhine Westphalia, is almost twice as likely to die prematurely as a citizen of Starnberg, an upscale district close to Munich and located between Lake Ammer and Lake Starnberg. In Starnberg a mere 18.4% of the population dies prematurely while the share of premature deaths in Herne is 34.3%.

We argue that these disparities in premature deaths result from imbalances in socioeconomic factors that determine premature mortality. This theoretical expectation corresponds with the data: The vast majority of variation in premature mortality across German districts and cities can be statistically explained by variation in socioeconomic factors. More importantly, the strong spatial patterns in actual premature mortality disappear when we look at residual premature mortality, that is, remaining premature mortality not accounted for by socioeconomic factors. We demonstrate that this holds for two different measures of premature mortality, namely for the share of the population that dies before the age of 75 (what we call the *premature mortality rate* for short) as well as for *potential years of life lost*, which we define as the total number of years lost relative to the age of 75 for those who die before reaching that age. The total sum of all potential years of life lost gives more weight to those who die very young and, naturally, exhibits greater variation than the premature mortality rate.

Others before us have analysed sub-national variation in premature mortality (e.g., Langford and Bentham 1996; Blackman and Dunstan 2010; Chen et al. 2010; Schofield et al. 2016), including in Germany (e.g., Wiesner and Bittner 2004, Sundmacher et al. 2012). Our main contribution is to demonstrate that the systematic spatial patterns in premature mortality found across Germany essentially disappear after socioeconomic factors that determine premature mortality have been taken into account. This original finding has major policy implications. Income, education, employment status and other socioeconomic factors affect lifestyle choices and behaviour as well as risk exposure and ultimately influence the health status of the population. Unless disparity in socioeconomic factors are directly tackled by a comprehensive set of economic and social policies going well beyond fiscal redistribution, the aim of ‘equivalent living conditions’ will remain elusive. Put differently, judged against the proxy measure of premature mortality, the politics of fiscal redistribution fall well short of a full convergence of ‘living conditions’ across Germany.

In section 2, we discuss the socioeconomic determinants of premature mortality. Section 3 demonstrates the systematic spatial clustering of premature mortality across Germany. In section 4, we describe spatial pattern recognition analysis as our method for estimating the extent to which socioeconomic factors can explain variation in premature mortality as well as drastically reduce and sometimes entirely eliminate the spatial patterns found in premature mortality. Section 5 concludes.

## Socioeconomic determinants of premature mortality: taking micro-level theories to the aggregate level

Many direct causes of premature death can be attributed to risky individual behaviour: Lung cancer predominantly results from smoking (Stewart and Kleihues 2003), coronary heart diseases are caused by high levels of fat in the nutrition and lack of personal fitness, and though 70% of Alzheimer’s disease cases seem to be caused by a genetic predisposition, the onset of the disease is influenced by how mentally and physically active someone is and the other 30% are associated with head injuries, depression and hypertension (Munoz and Feldman 2000). As the UK’s National Institute for Health and Care Excellence (2015: 2) has suggested, the most important direct causes of premature mortality, such as cancer, heart disease, stroke, respiratory and liver diseases, “are preceded by long periods of ill-health mostly caused by lifestyle related factors”.

The fact that the cumulative impact of individual behaviours ultimately triggers potentially fatal diseases renders premature deaths avoidable. However, the degree to which the diseases that result in premature mortality can be prevented varies. The following estimates suggest that, over all direct causes of premature mortality, between one third and two thirds of premature deaths could have been prevented. While Eurostat (2017) estimates that approximately a third of all premature deaths could have been avoided, the UK’s National Institute for Health and Care Excellence estimates that in England alone around two thirds of deaths of those aged 75 or below are avoidable. Estimates of premature deaths in the US suggests that about half of premature death are behaviour related and could have been prevented if individuals would not have smoked and were on a different diet (McGinnis 2013). Premature mortality in Starnberg is almost 50% lower than premature mortality in Herne. Since there is no systematic difference in the aggregate gene pool in the two places, this suggests that about 50% of the premature deaths in Herne would not have occurred if people had lived the life of the citizens of Starnberg and, of course, some of the premature deaths in Starnberg would have been avoidable, too.

Whilst the *direct causes* of premature mortality are predominantly the result of cumulative individual behaviours, premature mortality has socioeconomic *root causes*.^3^ This is because the behavioural triggers for the direct causes of premature mortality are not distributed evenly across society and instead systematically vary with socioeconomic factors such as income, education (Muennig 2010), professional occupation (blue collar versus white collar) and social status (Marmot and Allen 2014; Eberle et al. 2010). Liver diseases, respiratory diseases, cardiovascular diseases and cancer are up to two times more likely among the poor than among the rich (Department of Health 2003). Since robust evidence emerged that ‘smoking kills’, nicotine consumption has increasingly become a habit of the lesser educated parts of the population (Graham 2012). In fact, the fewer people who smoke, the larger the effect of income and education on nicotine consumption becomes. More educated individuals became increasingly less likely to start smoking and more likely to quit (Link 2008). The consumption of alcohol and a diet rich in fat are the most important triggers of liver diseases, with excessive alcohol consumption being the main cause of liver disease. An empirical study of the change in alcohol-related mortality after a significant reduction of alcohol taxes in Finland found that “a large reduction in the price of alcohol led to substantial increases in alcohol-related mortality, particularly among the less privileged, and in chronic diseases associated with heavy drinking” (Herttua et al. 2008: 1110). Bloomfield et al. (2000) find that alcohol abuse is much more common in Germans of low than high socioeconomic status.

Likewise, exposure to health hazards, independently of individual behaviour, also systematically varies with socioeconomic factors. For example, sectoral composition can directly influence premature deaths through industry-specific risks and accidents. Sector-specific employment may also indirectly affect health. For example, working in shifts, which is much more common in some sectors than in others, has been associated with a significantly higher propensity for coronary heart diseases (Knutsson 1988).

Differences in exposure to health hazards often reinforces the consequences of individual behaviour. For example, coronary heart diseases tend to be much more frequent in men with low income and low education than in any other group (Rose and Marmot 1981). Some studies have suggested that coronary heart diseases result from ‘job strain’—the combination of high job demands and low decision latitude, properties common in low status professions (Schnall et al. 1994). Similarly, chronic obstructive pulmonary disease is also more frequent in poorer parts of the population. The disease is characterised by airflow limitation associated with abnormal inflammatory response of the lungs to noxious particles or gases. Accordingly, chronic obstructive pulmonary disease develops not only as the consequence of a genetic predisposition—a rare hereditary deficiency of an anti-trypsin—but also because an individual has been exposed for extensive periods of time to dust, vapour and air pollution. The distribution of the disease thus correlates with socioeconomic factors because poorer and less educated individuals smoke more and are more likely to work in jobs that are exposed to dust, vapour and pollutants.

A second reason why premature mortality should systematically vary with socioeconomic factors at the local level is that poorer and more deprived local districts/cities have less money to spend on providing good health care as well as public goods such as parks and other recreational facilities conducive to good health (Macintyre et al. 1993). Yet, the vicious circle that goes from poverty and other socioeconomic deprivation, on the one hand, to illness, on the other, via this particular causal mechanism ought to be weak in (conservative) welfare states such as Germany (Hurrelmann et al. 2011). These states usually have a tax- or contribution-based health system that moderates the otherwise strong correlation between average income and health infrastructure at the local level.

## Spatial clustering in premature mortality rates

If all individuals were identical and had identical living conditions, premature mortality would occur by chance, but it would not systematically vary across space. While random processes would trigger some spatial variation in premature mortality at the district and cities level, these variations would not cluster strongly along spatial dimensions (authors). Instead, actual premature mortality clusters across space. This should not be surprising, since education and income, for example, influence behaviours at the individual level, so the aggregate level of education and income at the local level should also influence the spatial distribution of premature mortality. The rich and educated tend to live in different places than the poor and uneducated. Accordingly, socioeconomic factors cluster in space and, therefore, premature mortality also clusters in space. Areas with high per capita income and modern industries tend to have low premature mortality, while poor areas and the existence of ‘old’ and ‘dirty’ industries—especially of coal and steel—exhibit high premature mortality (van Doorslaer et al. 1997; Deaton and Paxson 2001).

In fact, if individual behaviour is not independent of the behaviour of other individuals, local variation in, for example, income could have a larger influence on premature mortality than variation in income across individuals. Individuals’ lifestyle choices to some extent depend on the lifestyle choices of their relatives, neighbours and peers. If these social contacts choose a healthier lifestyle, some of their peers will follow. The same logic also works in the opposite direction: when peers choose a less healthy lifestyle, others follow. Smoking, one of the more lethal habits, thus shows relatively strong social patterns. Since these social contacts usually depend on distance—closer individuals are more likely to interact than individuals further apart—lifestyle choices are not only socially stratified, but they also cluster geographically. In other words, spatial clustering in premature mortality in part derives from ‘learning processes’ and ‘social pressure’ at the local level.

Strong variations in premature mortality across space generate a political problem for democratic governments in welfare and transfer states like Germany. While it is convenient to blame premature mortality due to, for example, lung cancer on the individual who is ultimately responsible for their smoking habits, regional differences in premature mortality become a political issue—especially in a country in which equivalent living conditions are constitutionally mandated.

**Fig. 1:**
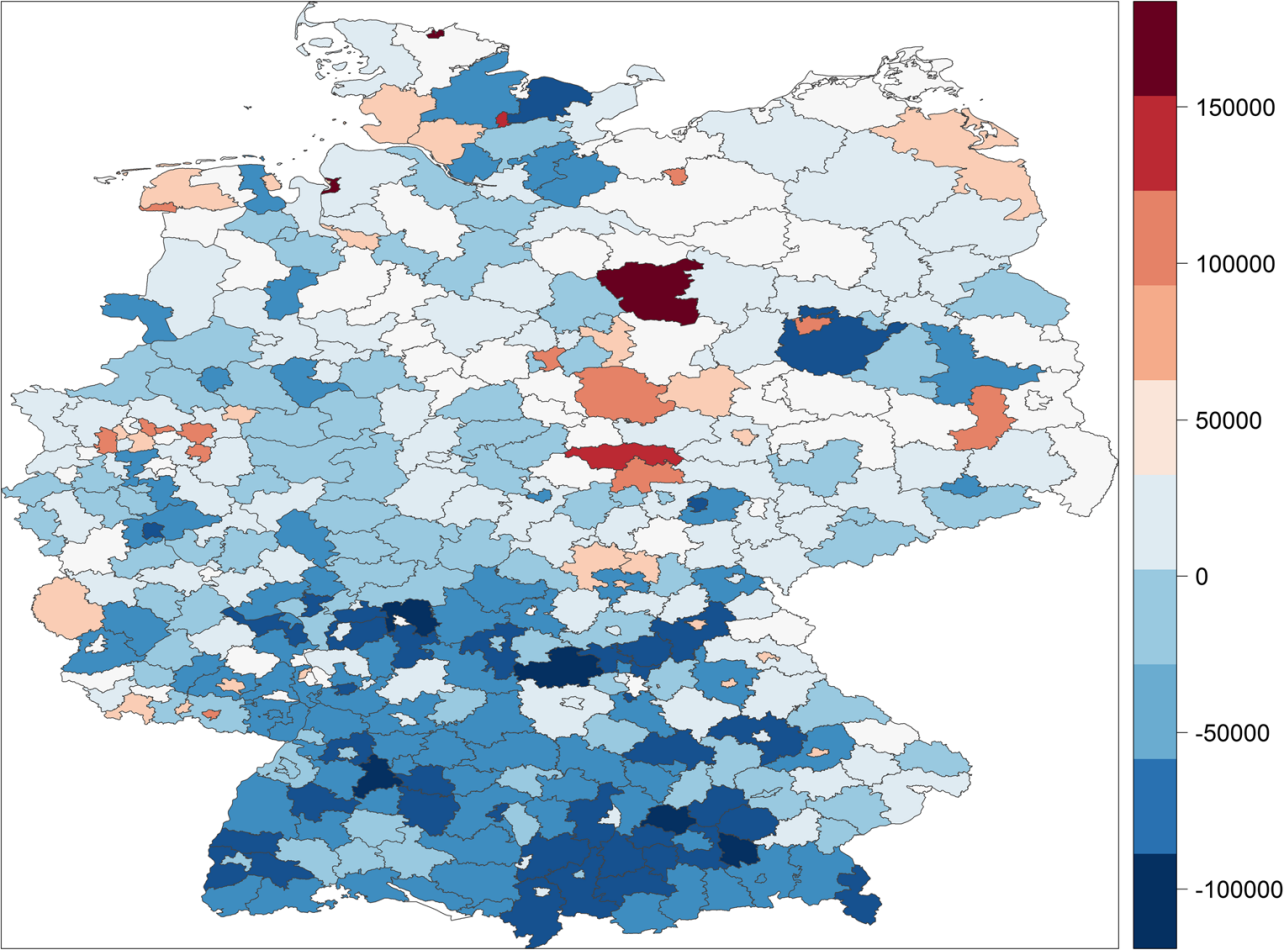
The distribution of years of potential life lost in German districts/cities

**Fig. 2:**
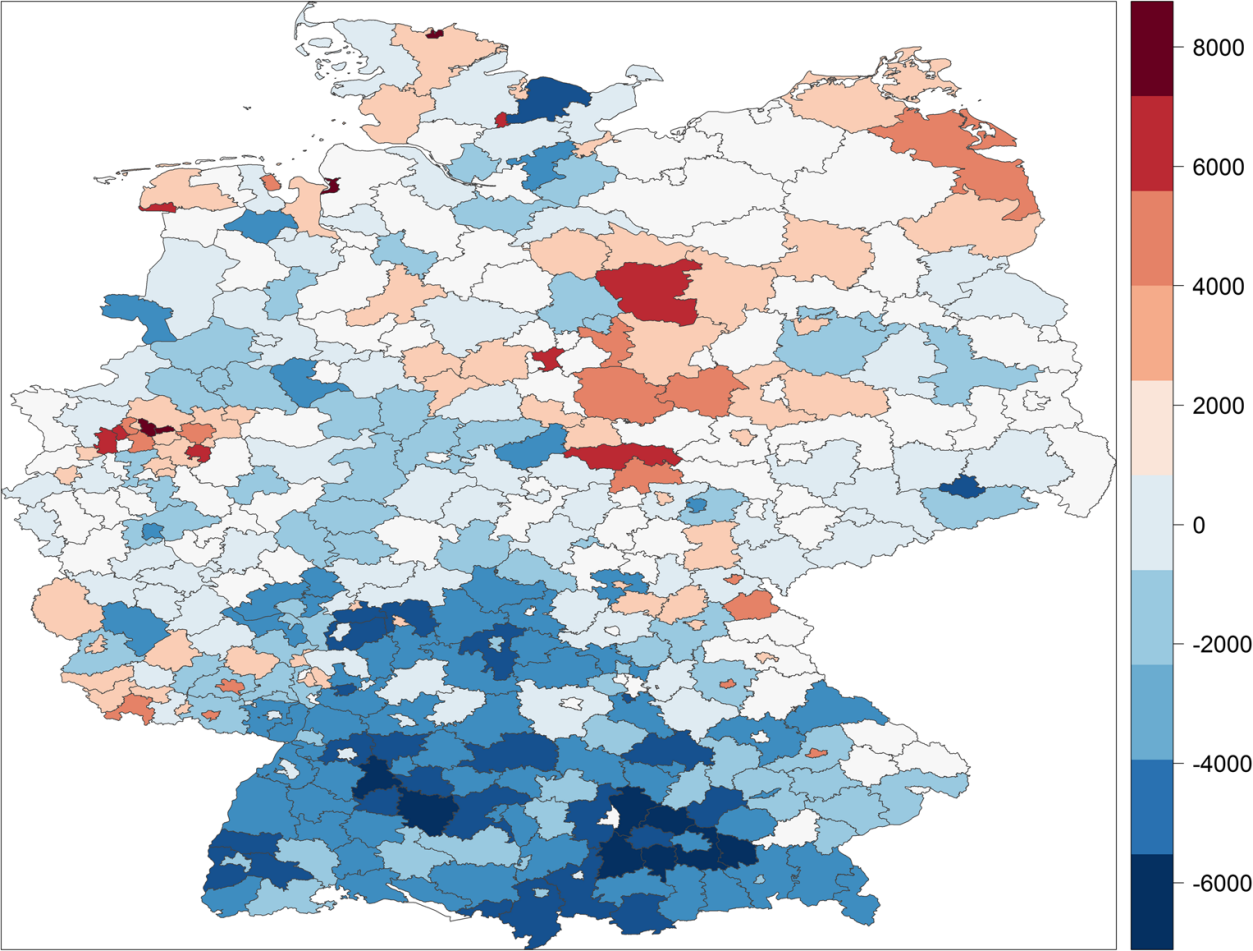
The distribution of premature mortality rates in German districts/cities

Figures 1 and 2 display the full distribution in potential years of life lost and premature mortality rates across German districts and cities, respectively. They map increasing degrees of above median (increasingly darker red) and below median (increasingly darker blue) premature mortality, with the scale in each map representing deciles of the two respective measures of premature mortality.

The German districts/cities with the highest rates of premature mortality are located in the old coal and steel cities of the Ruhr area, Herne, Gelsenkirchen and Duisburg, or in the ship industry cities, Bremerhafen, Flensburg and Emden, or in the rural, agricultural East German districts, Altmarkkreis, Kyffhäuserkreis and Salzlandkreis. The gap between the district with the highest rate of premature mortality, Herne, and the lowest rate, Starnberg, is astounding. While in Herne 34.4% of the population dies before the age of 75 and 48.1% dies before they reach the age of 80, in Starnberg only 18.4% of the population dies before the age of 75 and only 28.1% of the population dies before the age of 80. In other words, in Starnberg a significantly larger share of the population reaches the age of 80 than reaches the age of 75 in Herne.

**Fig. 3:**
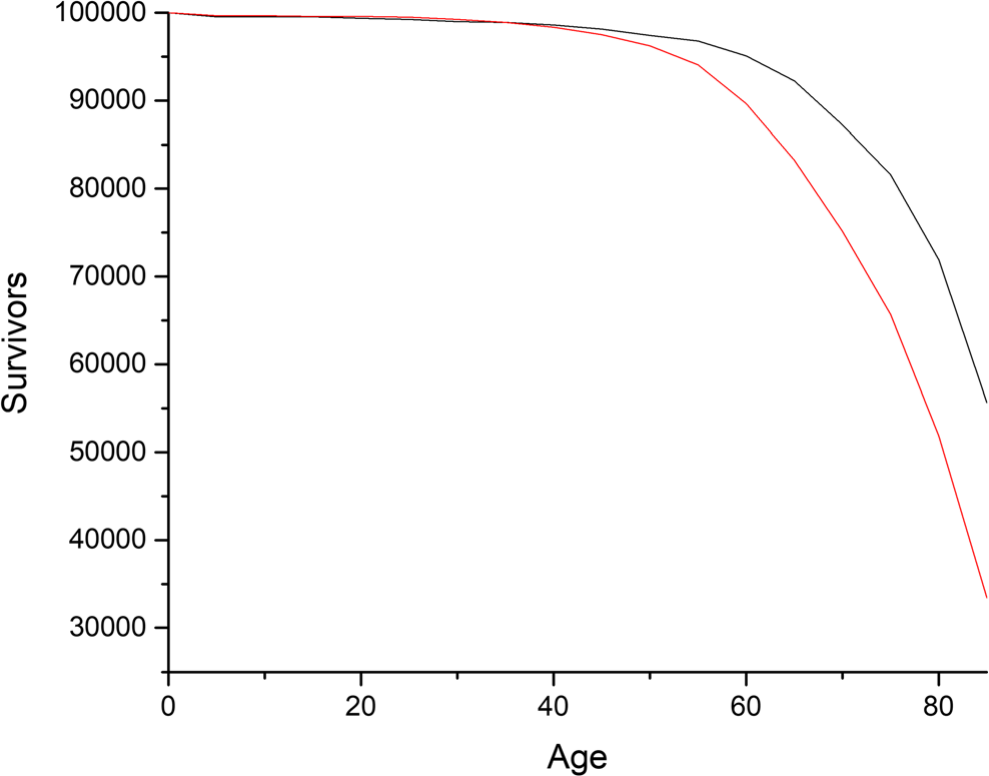
Survivor rates in Herne and Starnberg

Another way of displaying the stark difference between the two extremes of the German distribution is provided in Fig. 3, which shows the decline in survivors in an artificial population of 100,000 individuals in both Herne and Starnberg. While significant mortality in Herne already begins when our cohort reaches the age of 40, in Starnberg mortality remains low until our cohort turns 50. This 10-year gap declines only gradually after it reaches a maximum in the propensity to die when the artificial cohort reaches 75 years of age.

## Methods: spatial pattern recognition analysis of premature mortality

To analyse spatial patterns in premature mortality and the extent to which they are explained by spatial patterns in socioeconomic factors, we employ a spatial pattern recognition analysis. In a first step of this analysis, we estimate the extent to which systematic spatial patterns are detectable in premature mortality. Specifically, we regress premature mortality on five simple spatial measures: latitude, that is, the degree to which a district/city is located further north in Germany;^4^a dummy for East Germany; the clustering of premature mortality among geographically contiguous districts/cities; the clustering among geographically proximate districts/cities (defined as the inverse of Euclidean distance among districts); and urbanity, defined as population density of a district/city. Regressing premature mortality on these five spatial measures provides us with an estimate of the strength of the spatial patterns.

In the second step, we regress premature mortality on its socioeconomic determinants. This second step feeds into the final and most important step in which we use the predictions of the regression model from the second step to compute the residuals, which represent variation in premature mortality not explained by its socioeconomic determinants. We regress these residuals on the same five spatial measures. By comparing the coefficients from the first step of the spatial pattern recognition, which represent the strength of spatial patterns in premature mortality, with the coefficients from the third step of the spatial pattern recognition, which represent the remaining strength of spatial patterns in residual premature mortality that is unaccounted for by socioeconomic factors, we obtain an estimate of how much the spatial structure in premature mortality declines by accounting for spatial structure in its socioeconomic determinants.

As mentioned above, we define premature mortality as dying before the age of 75 and we use two measures, namely the premature mortality rate defined as the proportion of the population at the district/city level dying before the age of 75 and the total sum of potential years of life lost relative to the age of 75 for those dying prematurely. The bivariate correlation between the two measures is 0.9. For the first measure, we compute a standardised propensity of premature death in local authorities based on life tables we computed for 402 German districts and cities for the year 2014.^5^ These life tables allow us to compare the survival rate of an artificial cohort of 100,000 individuals in each district/city based on observed, that is, actual age-dependent probabilities of dying. Consider Germany as a whole: Starting with 100,000 individuals, 99,666 newborns reach their first birthday, 99,571 survive to experience their 10th birthday, 99,414 their 20th birthday, 96,925 reach their 50th birthday, and 88,247 individuals reach 65. At this point, mortality rates start to increase considerably. Only 74,392 reach the age of 75 and 42,404 individuals experience their 85th birthday. At this age, the information in the life tables ends. The premature mortality rate has a range from 18,423 to 34,307 with a mean of 25,604 and standard deviation of 2881.

The years of potential life lost measure is calculated as the sum of the number of standardised deaths at each cohort times the years that individuals died prematurely. For example in a given district/city the years of potential life lost would be the number of standardised deaths that occurred before the age of one multiplied by the 74 years that these children did not live (die prematurely) plus the standardised number of deaths between the ages of 1 and 5 multiplied by the 72 years they lost on average, plus the deaths between the ages of 5 and 10 multiplied by 67 years, and so on until the cohort that died between 70 and 75, which is multiplied by 2. This measure of premature mortality ranges from 199,814 to 502,682 with a mean of 321,634 and a standard deviation of 49,003.

We use five categories of socioeconomic variables: income and poverty; education; sectoral composition of the economy; socioeconomic status; and the depth and structure of employment.^6^ Specifically, we include mean household income and median workforce income at the district/city level as well as the average pension benefit of pensioners as proxies for disposable income. Per capita expenditures on social welfare and unemployment benefits as well as the total unemployment rate and the unemployment rate of women function as proxies for poverty. We further include information on the highest level of educational qualification of the population and the workforce,^7^ on the share of employment by economic sector and on the socioeconomic status composition of the workforce. Finally, we include the share of men and share of women in employment, the share of foreigners among the workforce as well as the ratio of part-time employment as measures of the depth and structure of employment. Since we use categories for educational achievement, categories of the sectoral composition of the economy and categories of socioeconomic status rather than continuous measures of these socioeconomic factors (e.g. years of schooling), we explicitly do not assume that the effect of, say, education on premature mortality is linear in the number of years of schooling, which would be highly implausible. Allowing for further non-linear effects by including second degree polynomial terms of our explanatory variables results in only a small increase in goodness-of-fit with the data and leaves our substantive findings unchanged (results not reported here).

Given that the socioeconomic factors included in our estimation model are not mutually independent of each other and some might represent the causal mechanism by which others exert their effect, we do not evaluate the point estimates of individual variables or their statistical significance. All we are interested in here is the combined explanatory power that socioeconomic factors jointly exert on premature mortality.

## Results

Table 1 presents the ordinary least square results of the first step of our spatial pattern recognition exercise, which identifies patterns in specified spatial dimensions in the outcome variable of interest, premature mortality. The first row presents results from regressing premature mortality on the latitude of districts/cities (‘Northness’). The next row displays results from a separate regression of premature mortality on an East Germany dummy variable. The next two rows are based on separate estimations using, respectively, average premature mortality in geographically contiguous and proximate districts/cities, capturing, respectively, contiguity clustering and proximity clustering. Finally, the last row reports results from regressing premature mortality on the population density of a district/city (‘urbanity’).

**Table 1:** Spatial patterns in observed premature mortality

	Years of potential life lost	Premature mortality rate
Northness	13,650	859.8
	(1231)	(70.73)
East Germany	33,636	1629
	(5987)	(356.4)
Contiguity	0.686	0.820
	(0.0628)	(0.0519)
Proximity	2.407	2.607
	(0.212)	(0.171)
Urbanity	11.52	0.999
	(3.579)	(0.207)

Note: Standard errors in parentheses

Table 1 confirms quantitatively what can be gleaned from Figs. 1 and 2. Districts/cities located further north tend to have higher premature mortality than those further south. Districts/cities located in the former German Democratic Republic of East Germany have systematically higher premature mortality than those located in former West Germany. Premature mortality has tended to converge since re-unification but our finding contradicts Wiesner and Bittner’s (2004) claim that they have almost fully converged. Premature mortality also clusters among geographically contiguous as well as geographically proximate districts/cities and is higher in more urban places. In substantive terms, districts/cities located in East Germany, for example, on average lose 1629 more people out of an artificial cohort of 100,000 (or to put it more simply, have a 1.63 percentage point higher premature mortality rate) and lose on average 33,636 more years of potential life than those in West Germany. With each degree of latitude, which varies from 47.6 to 54.8, the premature mortality rate increases by 860 (0.86 percentage points) and an additional 13,650 years of potential life are lost. These are large substantive effects given standard deviations of 2881 and 49,003, respectively, across Germany. The other coefficients could be similarly interpreted in terms of substantive effect sizes but our interest lies predominantly in the extent to which spatial patterns in socioeconomic factors reduce and potentially eliminate spatial patterns in premature mortality.

The ordinary least squares regression model in which we regress premature mortality on its socioeconomic determinants takes us to the second step of the spatial pattern recognition exercise. The results presented in Table 2 show that the socioeconomic characteristics of districts/cities account for between 62% (years of potential life lost) and 70% (premature mortality rate) of the cross-sectional variation in Germany. For a purely cross-sectional regression, this represents a truly large adjusted coefficient of determination and this very substantial overall explanatory power indicates the importance of the socioeconomic determinants for explaining inequality in premature mortality across German districts and cities.

**Table 2:** Results from regressing premature mortality on socioeconomic factors

	Potential years of life lost	Premature mortality rate
Educational qualification of population		
School leavers without qualification	547.5	−25.58
	(1043)	(54.83)
School leavers, secondary school qualification	54.23	4.024
	(280.7)	(14.76)
School leavers, university entrance qualification	−75.80	0.454
	(379.9)	(19.98)
Educational qualification of workforce		
Ratio of employees w/o professional qualification	2088	273.0
	(1749)	(92.00)
Ratio of employees with academic qualification	−2651	−131.2
	(2559)	(134.6)
Employment structure		
Share of women in employment	−1603	−151.4
	(826.4)	(43.46)
Share of men in employment	994.9	60.64
	(755.6)	(39.74)
Share of foreigners among employed	−2090	−161.0
	(810.7)	(42.64)
Share of part-time employees among employed	−2404	−171.3
	(1215)	(63.90)
Employment by economic sector		
Employment share primary sector	142.4	4.651
	(1238)	(65.11)
Employment share secondary sector	−407.9	−18.80
	(360.3)	(18.95)
Socioeconomic status		
Share of employed workforce, expert status	280.1	8.406
	(2908)	(153.0)
Share of employed workforce, specialist status	−196.0	33.62
	(1890)	(99.39)
Share of employed workforce, qualified status	1681	54.28
	(549.8)	(28.92)
Share of employed workforce, assistant status	443.6	28.72
	(1270)	(66.80)
Disposable income		
Average household income	−15.23	−0.979
	(10.57)	(0.556)
Median income of employed workforce	11.84	0.105
	(9.913)	(0.521)
Average pension income of retired population	−115.6	−6.999
	(42.95)	(2.259)
Poverty		
Unemployment ratio	9856	469.2
	(6587)	(346.4)
Unemployment ratio of women	−8006	−422.6
	(5660)	(297.7)
Per capita social welfare benefits	−3725	−189.3
	(1427)	(75.04)
Per capita unemployment benefits	12,362	711.5
	(3785)	(199.1)
Adjusted R-squared	0.62	0.70

*N* = 402. Robust standard errors in parentheses

To analyse to what extent the socioeconomic factors have eliminated the spatial patterns in premature mortality, Table 3 compares the estimates of the strength of correlation in the spatial dimensions in the observed values of premature mortality, as previously reported in Table 1 above, to the strength of correlation in the same spatial dimensions but this time in the residuals from the regression model of the second step of the spatial recognition exercise.

**Table 3:** Spatial patterns in observed premature mortality versus residuals

	Years of potential life lost			Premature mortality rate		
	Observed	Residuals	Percent decline	Observed	Residuals	Percent decline
Northness	13,650	419.7	97.0	859.8	21.23	97.5
	(1231)	(840.7)		(70.73)	(44.22)	
East Germany	33,636	−1716	100.0	1629	−160.7	100.0
	(5987)	(3714)		(356.4)	(195.2)	
Contiguity	0.686	0.0125	98.2	0.820	0.0938	88.6
	(0.0628)	(0.0428)		(0.0519)	(0.0350)	
Proximity	2.407	−0.0168	100.0	2.607	0.236	90.9
	(0.212)	(0.146)		(0.171)	(0.114)	
Urbanity	11.52	0.428	96.3	0.999	0.165	83.5
	(3.579)	(2.165)		(0.207)	(0.114)	

Standard errors in parentheses. Note that we truncate the decline when it exceeds 100%

We find that the decline in spatial pattern is higher for years of potential life lost than for premature mortality rates, but (with the exception of urbanity patterns in mortality rates) the decline exceeds 85% and is often 100% or very close to it. In other words, socioeconomic factors statistically explain spatial patterns in premature mortality almost completely. This of course implies that regional imbalances in Germany are not limited to premature mortality but also continue to exist for the socioeconomic factors that determine premature mortality.

**Fig. 4:**
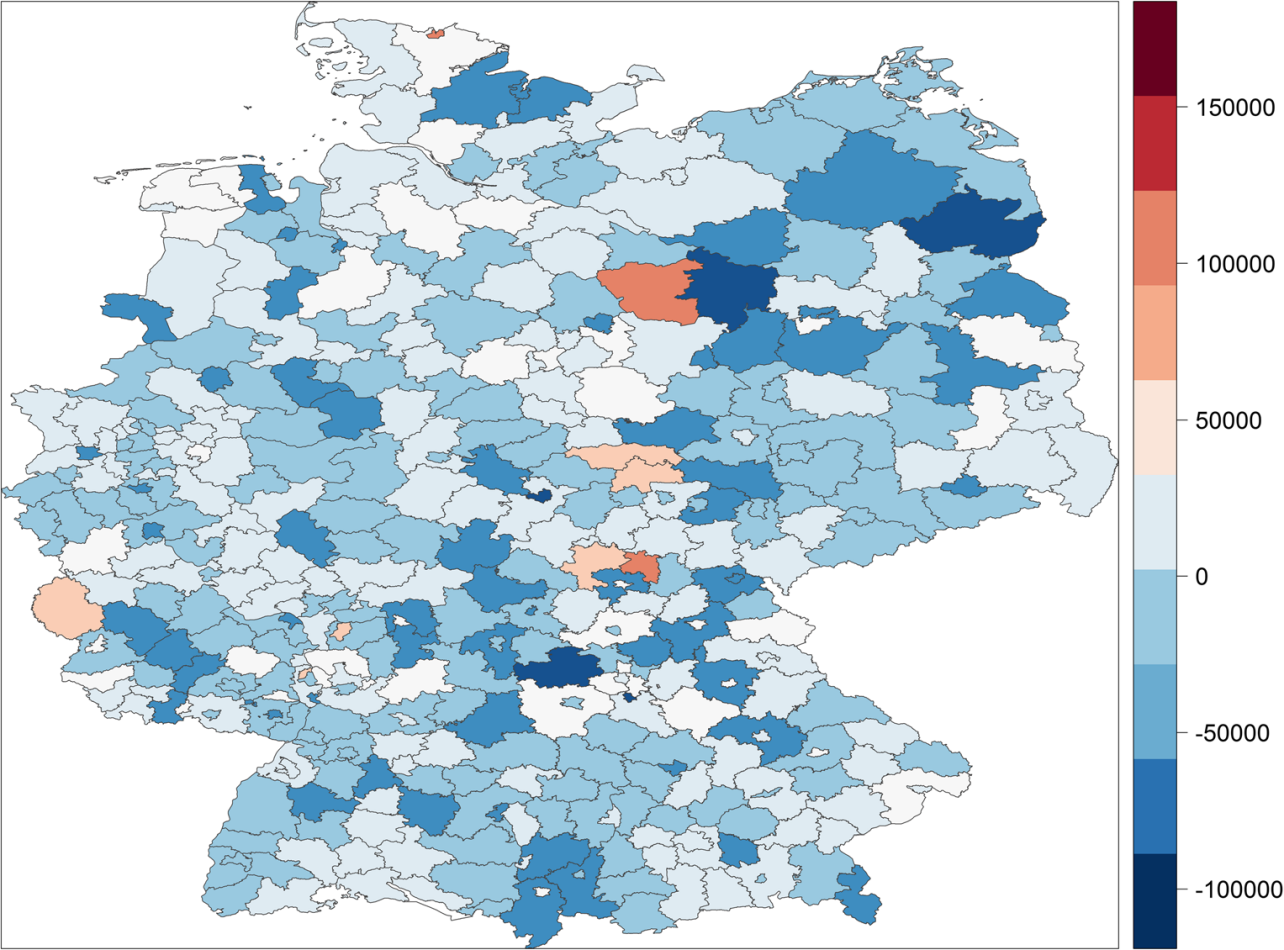
Unexplained variation in years of potential life lost

**Fig. 5:**
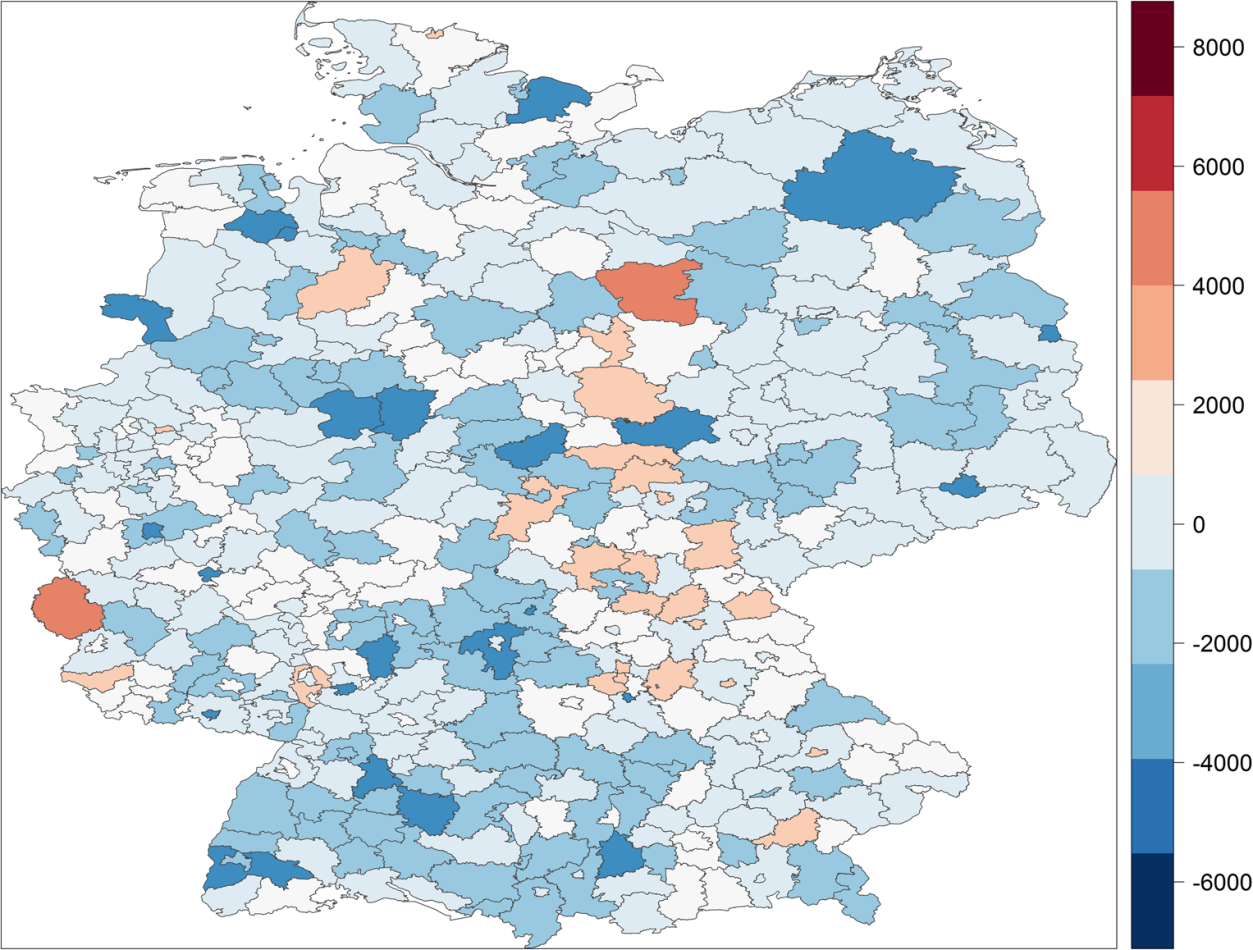
Unexplained variation in premature mortality rates

We can also visualise the ability of socioeconomic factors to explain the spatial patterns in premature mortality with the help of maps. Figures 4 and 5 map the residuals from our estimation models—that is, the variation in premature mortality unexplained by the socioeconomic explanatory variables—for years of potential life lost and the premature mortality rate, respectively.^8^ Clearly, the spatial patterns that were so prominent in Figs. 1 and 2 almost disappear when we map the residuals. There are still pockets of excessive morality even after structural disadvantages in socioeconomic factors have been taken into account, typically close to the former dividing border between West and East Germany, but on the whole Figs. 4 and 5 tend to show stochastic distribution across German districts and cities, whereas Figs. 1 and 2 exhibit systematic spatial patterns.

## Conclusion

Large disparities in premature mortality across German districts and cities continue to exist because disparities in socioeconomic factors that determine premature mortality continue to exist. Variations in average income, poverty and education, in the employment structure and in socioeconomic status create variations in living conditions—living conditions that persistently exert an influence on premature mortality.

These differences in premature mortality are not randomly distributed. Perhaps most importantly for the German political system, differences in premature mortality also continue to exist between Western and Eastern Germany though this is just one aspect of the systematic spatial patterns that can be found. The existence of these patterns demonstrates that the policy of fiscal equalisation does not suffice to generate equivalent living conditions. While we understand that equivalent living conditions do not imply identical living conditions, one cannot speak of equivalent living conditions when premature mortality in some German districts and cities is much higher than in others.

Our findings suggest that the political goal of equivalent living conditions requires much more than fiscal redistribution. Regarding premature mortality in particular, it also suggests that significant reductions in disparities across districts and cities cannot be achieved by targeted public health interventions alone. True, best practice smoking cessation services can result in a small reduction in lung cancer (Blackman and Dunstan 2010), and increasing the density and accessibility of physicians can result in a small reduction in overall mortality (Chen et al. 2010), particularly with respect to avoidable cancer such as breast cancer, colon, rectum and anus cancer (Sundmacher and Busse 2011). Yet, unless governments tackle and reduce inequalities in the socioeconomic determinants of premature mortality with a comprehensive set of economic, social and education policies, very large disparities will persist. This follows directly from our analysis, which demonstrated that the vast majority of variation of premature mortality is accounted for by socioeconomic factors and that the systematic spatial patterns found in actual premature mortality are fully or almost fully eliminated in residual premature mortality that remains beyond what the socioeconomic determinants can explain.

## Footnotes

1 Fiscal redistribution in Germany dates back to the Northern Federation, which established a moderate form of fiscal redistribution in 1867. Though the main purpose of this legislation was to reduce the dependence of the central government from the states, the legislation also balanced fiscal disparities across states and regions. In the Weimar Republic, the fiscal dominance of the states was abolished and replaced by a fiscal system in which the states became dependent on fiscal revenues collected by the central government. Despite these fundamental changes, Weimar’s fiscal order maintained the principle of fiscal harmonisation: states received a financial transfer from the state government that was largely proportional to the tax revenues raised in that state and the size of the population. This policy reduced fiscal disparities between richer and poorer states. In fact, the fiscal transfers guaranteed that no state had a per capita budget of less than 80% of the average per capita spending of all states.

2 75 is the most commonly used age threshold in definitions of premature mortality but our results hold for lower thresholds of 70 or 65.

3 See for example Link and Phelan (1995) for the theory of root causes applied to health issues.

4 Sundmacher and Busse (2011) have previously noted a north-south gradient in avoidable cancer deaths in Germany.

5 Data were obtained from the Regional Database of Germany (Regionaldatenbank Deutschland) https://www.regionalstatistik.de/genesis/online last accessed in August 2016.

6 Data were obtained from the Bundesinstitut für Bau- Stadt- und Raumforschung website (http://inkar.de/Default) last accessed in January 2017.

7 For comparability, these characteristics are measured as a proportion of the relevant district/city population or workforce (e.g. the share of the local population that has left school with a university entrance qualification).

8 For comparability, Figs. 4 and 5 use the same scales as Figs. 1 and 2, respectively.
